# Investigating associations between hearing, cognition, and social isolation using the Hertfordshire Ageing Study

**DOI:** 10.3389/fpubh.2025.1658569

**Published:** 2025-10-17

**Authors:** Nisha Dhanda, Amanda Hall, James Martin, Helen Pryce

**Affiliations:** ^1^Department of Applied Health Sciences, College of Medicine and Health, University of Birmingham, Birmingham, United Kingdom; ^2^Department of Audiology, College of Health and Life Sciences, Aston University, Birmingham, United Kingdom

**Keywords:** epidemiology, hearing loss, cognition, social isolation, older adults, ageing

## Abstract

**Objectives:**

The aim of the study was to investigate whether hearing threshold separately predicts cognitive score and social isolation score 10 years later by using the Hertfordshire Ageing Study (HAS) data.

**Methods:**

The Hertfordshire Ageing Study (HAS) is a longitudinal cohort study that measures hearing via pure tone audiometry at two timepoints, and social isolation and cognition variables at the second timepoint. Linear regression was implemented for both objectives using an unadjusted model, a model controlling for age and gender, and a model controlling for all confounders (sociodemographic, lifestyle, and clinical characteristics) relating to the exposure and outcome variables. For interpretability, coefficients were expressed as the expected change in outcome per doubling of hearing threshold.

**Results:**

A total of 231 and 254 participants were included in the final analyses. Over 10 years, hearing thresholds worsened by an average of 10.5 dB. Higher hearing thresholds were associated with lower MMSE scores (*β* per doubling of hearing = −1.02, 95% CI –2.07–0.03) and with lower social isolation scores (*β* per doubling = −0.37, 95% CI –1.40–0.66). Although these associations were not statistically significant, the confidence intervals suggest that small but potentially meaningful effects cannot be excluded.

**Conclusion:**

The lack of evidence of an association despite strong theoretical evidence may be due to selection bias within the overall cohort study and the sensitivity of the outcome measures used for social isolation and cognition.

## Introduction

The consequences of unmanaged age-related hearing loss are far-reaching (1). In recent years, the connection between hearing loss and cognition has been increasingly investigated (2–4). Hearing loss in midlife has been identified as the most significant modifiable risk factor (alongside high LDL cholesterol) to dementia in later life (5). Accordingly, identifying strategies to prevent or delay the onset of dementia is a central priority within healthy ageing initiatives. Despite this interest, the pathways underlying the hearing–cognition relationship remain poorly understood. Two main hypotheses have been proposed. The first suggests a shared neuropathological origin, such as neurodegeneration or chronic inflammation (6). The second, the “cascade” hypothesis, posits that hearing loss initiates downstream changes that accelerate cognitive decline (7). Social isolation is frequently cited as one such factor because it is independently associated with both hearing loss (8, 9) and cognitive decline (10). However, empirical evidence for this pathway remains limited and inconsistent.

A systematic review of longitudinal studies found that only one out of fifteen formally tested social isolation as a mediator, and it reported no evidence to support this role (11, 12). One reason for this gap may be the inconsistent and often simplistic measurement of social isolation across studies. Some have relied on marital status or frequency of contact as proxies (12), while others have used single-item classifications (13). These approaches fail to capture the multidimensional nature of social isolation, which encompasses structural, functional, and qualitative aspects of social relationships (14, 15). More comprehensive tools, such as the Medical Outcomes Survey (MOS) Social Support Survey, assess multiple domains, such as emotional, tangible, and informational support, and may provide a more valid measure (16).

Prior studies have also differed in their methodological rigor. For example, one study (17) reported that loneliness and social isolation mediated the association between self-reported hearing impairment and episodic memory over 10 years, while another (13) found only partial support using a simplified measure of social isolation. Both studies relied on self-reported hearing loss and narrow cognitive assessments, which may limit causal inference and generalisability. These limitations highlight the need for research using objective hearing measures, validated social isolation tools, and broader cognitive assessments.

The Hertfordshire Ageing Study (HAS) addresses several of these gaps. It includes objective audiometric measures of hearing, a validated multidimensional measure of social isolation, and a general cognitive assessment, all within a longitudinal design where hearing was assessed prior to social isolation and cognition. While the current analyses do not formally test mediation, they examine two key associations that provide foundational evidence for future mediation models:

1. Does hearing threshold predict cognitive score 10 years later?
2. Does hearing threshold predict social isolation score 10 years later?

By clarifying these long-term associations using robust measures, this study contributes to understanding whether social isolation may be a relevant factor in the hearing–cognition pathway.

## Methods

### Study design

The Hertfordshire Ageing Study (HAS) was a birth cohort study whose principal objective was to examine life course influences on healthy ageing (18). There were 6,803 live singletons born in North Hertfordshire between 1920 and 1930. With the help of the National Health Service Central Register, 1,428 who still lived there in 1995 were traced, and 824 (58%) of the traced people agreed to a home interview. After the interview, 717 men and women attended a clinic for detailed characterization of ageing in a range of measures, such as hearing. Regular inter- and intra-observer reliability assessments were conducted throughout the fieldwork to ensure consistency and comparability of measurements (18). These procedures strengthened the methodological rigor of the study by minimizing measurement error and enhancing the validity of the data.

### Participants

**Figure 1:**
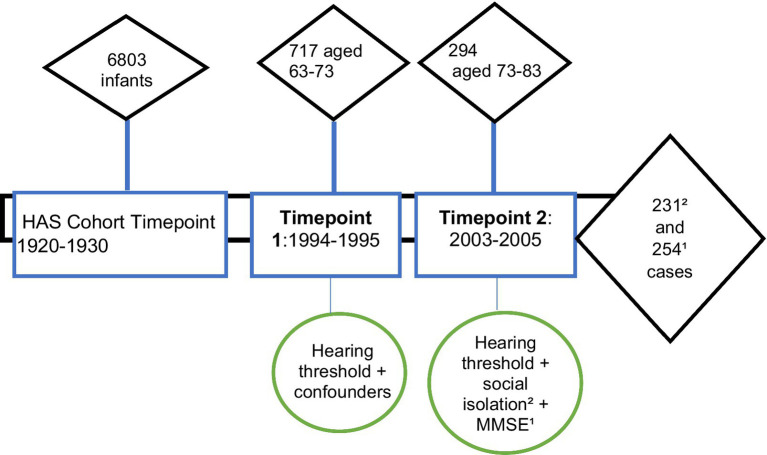
Hertfordshire Ageing Study timepoints showing participant numbers and relevant variables measured. Timepoint 1 = Timepoint 1; Timepoint 2 = Timepoint 2; MMSE = mini mental state examination.

In the Hertfordshire Ageing Study (HAS), participants had data collected at two points in time—roughly in 1994–5 and 2003–5. The average age of the study participants was 67 years at timepoint 1 and 76 years at timepoint 2. The first HAS follow-up (timepoint 1) was conducted in 1994–95 when the participants ranged in age from 63 to 73 years (mean 67). This consisted of 717 participants who underwent pure tone audiometry (0.5–4 kHz) at timepoint 1 and 294 at timepoint 2. Attrition was high and resulted in a small analytical sample. There were 254 complete cases for hearing and cognition, and 231 complete cases for hearing and social isolation, which make up the analysis sample (Figure 1).

### Hearing threshold

Hearing thresholds were assessed using pure tone audiometry. Trained researchers assessed hearing in 717 individuals at timepoint 1 and 294 individuals at timepoint 2. Audiometric thresholds were measured by air conduction at four frequencies (500, 1,000, 2,000, and 4,000 Hz). The average hearing threshold was the mean threshold value at 500, 1,000, 2,000, and 4,000 Hz by air conduction for the worse hearing ear, with higher values indicating more hearing loss. The British Society of Audiology Recommended Procedures defines normal hearing as having a hearing threshold of 20 dB or below (19). These clinical recommendations, followed by clinicians throughout the UK, are a valid way to distinguish between “normal” and “abnormal” hearing thresholds.

### Cognitive outcomes

Cognition was assessed using the Mini-Mental State Examination (MMSE) (20) at timepoint 2. This 30-point cognitive screening tool assesses the following cognitive functions: orientation, registration, attention and calculation, recall, language, and copying. The MMSE allows for a maximum score of 30. A score of less than 25 is typically seen as abnormal and indicative of possible cognitive impairment.

The MMSE, widely used in clinical practice as a dementia screening tool due to its ease of administration and comprehensive assessment of cognitive domains, is the most popular choice for assessing cognitive status (11), despite its potential disadvantage for individuals with unmanaged hearing loss due to the verbal nature of some items, requiring adequate auditory function.

### Social isolation outcome

Social isolation was assessed using the MOS Social Support Survey (16) at timepoint 2. The survey was made up of four functional support scales (emotional/informational, tangible, affectionate, and positive social interaction) and the construction of an overall functional social support index. Eight self-reported questions related to social isolation were asked, with answers given on a Likert scale from 1 (complete isolation) to 5 (no isolation). Questions included: someone you can count on to listen to when you need to talk; someone to confide in or talk to about yourself or your problems; someone to share your most private worries and fears; someone who understands your problems; someone who shows you love and affection; someone to do something enjoyable with; how often do you see children; how often do you see neighbors? Each self-reported answer was rated from 1 to 5 on a Likert scale, with a lower score indicating greater social isolation. Social isolation was measured at timepoint 2 only. A notable consideration is that the MOS survey measures perceived availability of support rather than actual social contact or network size. This means someone may feel supported even if they have few social interactions, which can underestimate social isolation.

### Confounders

Confounding variables, chosen based on the available data and the existing literature, were self-reported at timepoint 1 and were assumed to be unchanged at timepoint 2. These included: age, gender, social class, smoking status, alcohol consumption, marital status, education status, and clinical diagnoses (angina, stroke, heart attack, high blood pressure, type 2 diabetes, or depression). Age was regarded as a continuous variable, while all others were regarded as categorial. Self-reported social class was categorized into either professional, managerial, technical, and non-manual, or manual, partly skilled, and unskilled. This referred to a person’s own social class or their husband’s, if ever married.

### Statistical analysis

The characteristics of the participants were appropriately summarized using counts and percentages for categorical variables, and continuous variables were summarized using either mean and standard deviation or median and inter-quartile range, depending on the data distribution. We additionally compared the characteristics of those included in the study with the non-responders using Chi-squared and Kruskal–Wallis tests.

For each outcome, to understand its relationship with hearing threshold, we fitted three linear regression models: an unadjusted model, a partially adjusted model (with age and sex), and a fully adjusted model (age, sex, social class, smoking status, number of alcoholic units drunk per week, marital status, years of education, diagnosed angina, stroke, heart attack, high blood pressure, type 2 diabetes, or depression).

The hearing threshold level at timepoint 1 was negatively skewed, so we have summarized it using the median and interquartile range. For the regression analysis, a log transformation was used to better meet the assumptions of linear regression. In this model specification, the regression coefficient represents the change in the outcome for a one-unit increase in ln(hearing threshold). For interpretability, we also present results expressed as the expected change in the outcome per doubling of the hearing threshold. This is obtained by multiplying the coefficient by ln(2) because doubling the hearing threshold increases ln(hearing threshold) by ln(2). A complete case analysis was used, ensuring that only comprehensive datasets were included in the analysis (21). This approach was chosen to maintain consistency across models and avoid introducing assumptions required for imputation. However, this reduced the analytic sample size and may have affected statistical power. The final sample size after exclusions was 231 for hearing and social isolation and 254 for hearing and cognition.

## Results

At timepoint 2, which was conducted in 2003–2005, there was high attrition, resulting in 294 participants who had completed hearing measures at both timepoints. There were 121 participants who had died between timepoint 1 and timepoint 2, and 409 participants who did not respond, declined to participate in timepoint 2, or were untraced. The average age of the study participants was 67 years at timepoint 1 and 76 years at timepoint 2. The majority (58.8%) were men and were current smokers (10.2%) or ex-smokers (53.1%) (Table 1). Most of the participants were married (74.1%), drank 10 units or less of alcohol per week (49%), or no alcohol (34%). Of the clinical diagnoses, high blood pressure was the most prevalent (30.2%), with depression (13.3%) and heart attack (9.6%) the next most prevalent.

**Table 1:** Population characteristics of included sample compared with non-responders.

Variables	*N* (294)	Mean or Percentage	SD or IQR	Non-responders – Died (121)	Non-responders – Declined/Untraced (409)	*p*-value*
Exposure						
Average hearing threshold right ear T1 (dB)	293	27.50 (median)	17.50 (IQR)	(*n* = 120) 23.75	(*n* = 308) 22.50	0.15
Average hearing threshold left ear T1 (dB)	293	20.00 (median)	13.75 (IQR)	(*n* = 120) 30.00 (median)	(*n* = 308) 26.25 (median)	0.031
Average hearing threshold right ear T2 (dB)	254	38.02 (median)	15.68 (IQR)	(*n* = 120) 31.25 (median)	(*n* = 308) 27.50 (median)	0.034
Average hearing threshold left ear T2 (dB)	254	29.23 (median)	12.66 (IQR)	(*n* = 120) 22.50 (median)	(*n* = 308) 21.25 (median)	0.07
Maximum change in hearing T1-T2 (dB/year), median [Inter quartile range (IQR)]	253	6.9	1.17			
Cognitive outcome						
MMSE score (Timepoint 2)	254					
Normal 25+	236	92.90%				
Impaired ≤24	18	7.10%				
Demographic and lifestyle characteristics						
Age at follow-up 1 (years)	294	66.97 (median)	3.55 (IQR)	67.96 (median)	(*n* = 302) 67.39 (median)	0.006
Age at follow-up 2 (years)	294	76.43/76.30 (median)	2.22/3.70 (IQR)			
Gender						
Male	173	58.80%		81 (66.9%)	205 (50.1%)	0.002
Female	121	41.20%		40 (33.1%)	204 (49.9%)	
Smoking status at follow-up 1						
Never	108	36.70%		28 (23.1%)	150 (36.7%)	0.001
Ex-smoker	156	53.10%		69 (57.0)	184 (45.0%)	
Current smoker	30	10.20%		24 (19.9%)	75 (18.3%)	
Alcohol units per week at follow-up 1						
Non-drinker	100	34.00%		42 (34.7%)	167 (40.8%)	0.38
≤10 units	144	49.00%		56 (46.3%)	177 (43.3%)	
>11 units	50	17%		23 (19%)	65 (15.9%)	
Marital status at follow-up 1						
Single, Divorced, Widowed	76	25.90%		37 (30.6%)	127 (31.1%)	0.303
Married	218	74.10%		84 (69.4%)	282 (68.9%)	
Own social class^ at follow-up 1 (4 missing)				120	405	
I	133	45.90%		46 (38.3%)	161 (39.8%)	
II	157	54.10%		74 (61.7%)	244 (60.2%)	0.196
Years of further education (241 missing)				18	37	
1–10 years	50	94.30%		17 (94.4%)	36 (97.3%)	0.789
11–20 years	3	5.70%		1 (5.6%)	1 (2.7%)	
Diagnosed heart attack (2 missing)				121	402	
Yes	28	9.60%		15 (12.4%)	28 (7.0%)	0.143
No	264	90.40%		106 (87.6%)	374 (93.0%)	
Diagnosed angina (2 missing)				121	403	
Yes	23	7.90%		15 (12.4%)	51 (12.7%)	0.116
No	269	92.10%		106 (87.6%)	352 (87.3%)	
Diagnosed high blood pressure (3 missing)				119	403	
Yes	88	30.20%		44 (37.0%)	128 (31.8%)	0.411
No	203	69.80%		75 (63.0%)	275 (68.2%)	
Diagnosed stroke				119	406	
Yes	2	0.70%		7 (5.9%)	15 (3.7%)	0.008
No	292	99.30%		112 (94.1%)	391 (96.3%)	
Type 2 diabetes (15 missing)				111	288	
Yes	15	5.40%		18 (16.2%)	25 (8.7%)	0.003
No	264	94.60%		93 (83.8%)	263 (91.3%)	
Low mood/depression				121	408	
Yes	39	13.30%		19 (15.7%)	59 (14.5%)	0.796
No	255	86.70%		102 (84.3%)	349 (85.5%)	
Emotional/Informational support	232	3.97 (0.96)				
Tangible support	233	3.96 (1.20)				
Affectionate support	233	4.16 (1.11)				
Positive social interaction	231	4.05 (1.06)				
Additional item	232	3.92 (1.16)				
Overall support index	231	7.93 (2.04)				

When comparing non-responders (those who died before timepoint 2 and those who declined/untraced) to the study participants, the non-responders were generally: older age, male gender, worse average hearing threshold, current smoking status at timepoint 1, drinking >11 alcohol units per week, a marital status of single/divorced/widowed, lower social class, diagnosis of stroke, and type 2 diabetes.

The median worse-ear hearing threshold (referred to as “hearing threshold”) was 27.50 dB HL at timepoint 1, worsening to 38.02 dB HL at timepoint 2. This represents an average decline of 10.5 dB, which is clinically meaningful because decibels are measured on a logarithmic scale. A 10-dB increase corresponds to roughly a doubling in perceived loudness. In practical terms, this means that sounds that were previously audible at the level of a soft whisper (around 25–30 dB) would need to be as loud as a quiet conversation (around 35–40 dB) to be detected. The rate of change in hearing thresholds between the two assessment points exhibited considerable inter-individual variability, with values ranging from a maximum of 6.9 dB per year to a mean of 0.76 dB per year (SD = 1.17). This variation indicates heterogeneity in the progression of hearing decline across participants.

### Hearing loss and cognition

**Figure 2:**
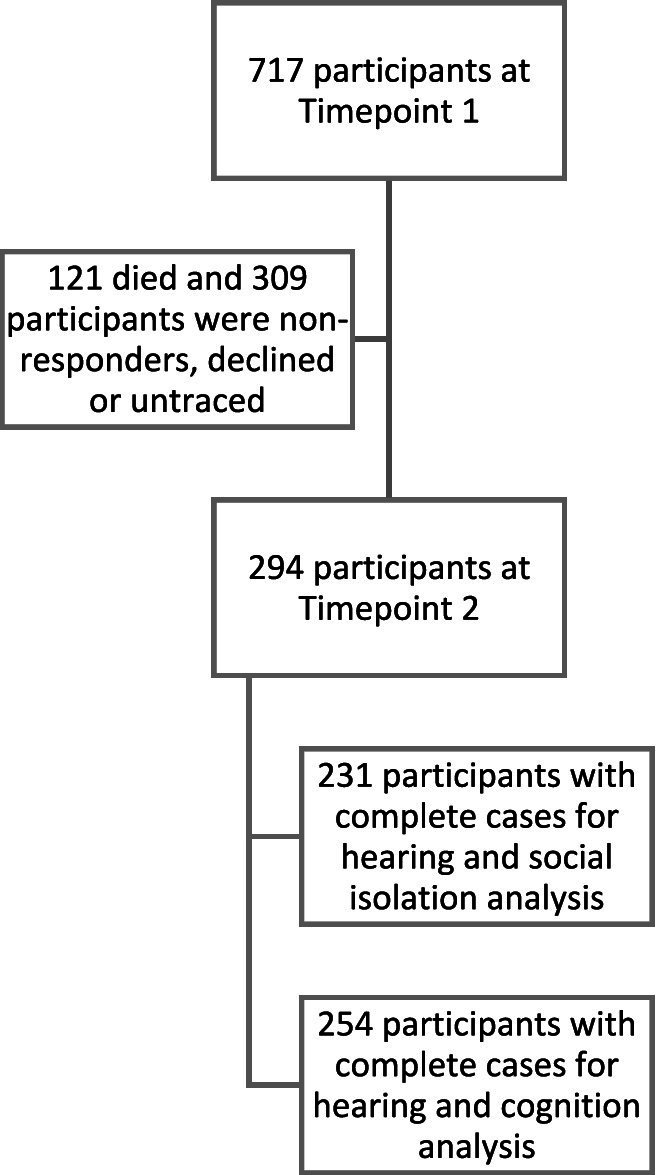
Flow diagram showing the number of participants at each timepoint and the number of participants included in the analysis.

Of the 294 in the final included sample, 254 participants had completed the MMSE data and 231 had completed the social isolation survey (Figure 2).

Among 254 participants with MMSE data, 92.9% scored ≥25 at timepoint 2. Across all models, hearing threshold was associated with a lower cognitive score, though these associations were not statistically significant. In the unadjusted model, a one-unit increase in ln(HL) was associated with a 1.48 decrease in MMSE (95% CI –2.99–0.04). When rescaled to a doubling of hearing threshold, this corresponds to a 1.02 decrease in cognitive score (95%CI: −2.07–0.03). Adjustment for age and sex attenuated the association to a − 0.74 change in MMSE per doubling in HL threshold (95%CI –1.79–0.31), and in the fully adjusted model, the estimate was −0.64 (95%CI –1.71–0.43) per doubling in HL. The decrease in effect size with increased adjustment suggests the influence of the aforementioned confounding variables on observed associations (Table 2).

**Table 2:** Regression coefficients for hearing threshold (exposure) at timepoint 1 and cognitive scores (outcome) at timepoint 2.

Linear regression models	Change in MMSE per 1-unit increase in ln(HL) (95%CI)	Change in MMSE per doubling of dB HL	*p*-value
Model 1 – Univariable	−1.476 (−2.992, 0.039)	−1.02 (−2.07, 0.03)	0.056
Model 2 – Adjusted for age and sex	−1.067 (−2.586, 0.453)	−0.74 (−1.79, 0.31)	0.168
Model 3 – Fully adjusted	−0.923 (−2.471, 0.625)	−0.64 (−1.71, 0.43)	0.241

HL = hearing threshold (dB HL). The predictor in all models was the natural logarithm of hearing threshold [ln(HL)]. A one-unit increase in ln(HL) corresponds to multiplying the hearing threshold itself by e (≈2.72). To make the results easier to interpret, we also present the effect of a doubling of hearing threshold. This is obtained by multiplying the reported coefficient by ln(2) (≈0.693), because an increase of ln(2) in ln(HL) is exactly what happens when the hearing threshold doubles. Negative values mean that worse hearing (higher HL) is associated with lower cognitive score.

### Hearing loss and social isolation

For 231 participants with social isolation data, associations with hearing thresholds were small and non-significant across all models. In the unadjusted model, a one-unit increase in ln(hearing threshold) was associated with a 0.53 decrease in social isolation score (95%CI –2.02–0.96). Expressed as a doubling in hearing threshold, this equates to a − 0.37 change in social isolation score (95%CI: −1.40–0.66). After adjusting for age and sex, the association was −0.45 per doubling in HL (95%CI –1.50–0.60), and −0.41 (95%CI: −1.44–0.62) in the fully adjusted model. These results indicate no consistent evidence of an association between hearing threshold and social isolation in this cohort. In other words, the observed estimates might be due to random variation rather than a true relationship (Table 3).

**Table 3:** Regression coefficients for hearing threshold at timepoint 1 and social isolation scores at timepoint 2.

Linear regression models	Change in isolation score per 1-unit increase in ln(HL) (*β*, 95% CI)	Change in isolation score per doubling of HL (*β*, 95% CI)	*p*-value
Model 1 – Univariable	−0.530 (−2.019, 0.959)	−0.37 (95%CI: −1.40–0.66)	0.483
Model 2 – Adjusted for age and sex	−0.651 (−2.172, 0.869)	−0.45 (95%CI: −1.50–0.60)	0.399
Model 3 – Fully adjusted	−0.595 (−2.083, 0.893)	−0.41 (95%CI: −1.44–0.62)	0.431

HL = hearing threshold (dB HL). The predictor in all models was the natural logarithm of hearing threshold [ln(HL)]. A one-unit increase in ln(HL) corresponds to multiplying the hearing threshold itself by e (≈2.72). To make the results easier to interpret, we also present the effect of a doubling of the hearing threshold. This is obtained by multiplying the reported coefficient by ln(2) (≈0.693) because an increase of ln(2) in ln(HL) is exactly what happens when the hearing threshold doubles. Negative values mean that worse hearing (higher HL) is associated with a social isolation score.

## Discussion

This study investigated the longitudinal associations between hearing threshold and subsequent cognitive function, as well as between hearing threshold and social isolation, in community-dwelling older adults. Over a 10-year period, the cohort’s average hearing threshold declined by 10.5 dB. Contrary to previous research (3, 22, 23), our analysis identified no statistically significant associations between hearing loss and cognitive performance or between hearing loss and social isolation. However, the relatively small sample size of the study means we were likely underpowered to detect small-to-moderate effects. Future studies need to be of sufficient sample sizes to ensure these small effect sizes do not go undetected. Although not statistically significant, the associations were inverse, with effect estimates and confidence intervals aligning with trends reported in prior studies (24, 25).

Given the sample size, it is important to interpret these results in the context of the confidence intervals, rather than relying solely on statistical significance. For cognition, the 95% CI for the effect of a doubling of hearing threshold (−2.07–0.03) suggests that worse hearing could be associated with up to a two-point lower MMSE score, which would be clinically important, though the interval also includes no effect. For social isolation, the confidence intervals span both modest decreases and increases in social isolation score, indicating that the true association could be small in either direction. While *p*-values indicate a lack of statistical significance, the confidence intervals for MMSE suggest that clinically meaningful effects cannot be ruled out. Several factors may account for these findings here. A recent cohort study similarly reported no significant effect of hearing loss on cognitive decline after adjustment for age (26), highlighting the importance of controlling for confounding variables and employing outcome measures sensitive to subtle, long-term changes. The observed hearing decline of 10.5 dB over 10 years is relatively modest and may not be clinically meaningful, as declines of 15 dB or more at specific frequencies are typically considered significant in older adults (27, 28). If hearing changes were insufficient to affect daily functioning, null associations with cognition and social isolation would be expected. Furthermore, most participants maintained MMSE scores within the normal range, suggesting minimal cognitive impact from the observed hearing decline.

Our findings diverge from much of the existing literature, and several explanations are plausible. First, our analysis controlled for thirteen potential confounders, including age. Additionally, publication bias may contribute to the predominance of significant findings in the literature. Null results such as ours provide valuable evidence in a field where non-significant findings are under-represented, potentially distorting perceptions of the true relationship between hearing loss and cognition (29, 30).

The use of complete-case analysis introduces potential selection bias. Attrition between baseline and follow-up was substantial, and non-responders were older, had poorer hearing, and exhibited more comorbidities, which were the group most likely to demonstrate associations between hearing loss and cognitive or social outcomes. This “healthier survivor” effect may have attenuated observed associations, warranting cautious interpretation of the null findings. Our models also assumed stability in covariates such as marital status, health conditions, and social class over the 10-year interval. In reality, these factors likely changed and could influence both hearing and cognitive/social outcomes. The inability to account for time-varying confounding represents a key limitation and may have contributed to the null associations observed. Our relatively small sample size compared with previous studies (3, 22) likely reduced statistical power, limiting our ability to detect associations due to underpowering. We used the MMSE to assess cognitive performance; however, despite its widespread use, the MMSE is primarily a screening tool for cognitive impairment and has limited sensitivity to subtle changes associated with normative aging. This limitation is evident in our sample, where 92.9% of participants scored above the commonly used cutoff of 25, suggesting a ceiling effect that may have obscured associations. A more comprehensive psychometric battery would likely provide greater sensitivity to age-related cognitive variation. Similarly, epidemiological measures of social isolation may not capture nuanced patterns as effectively as individual-level observations. Future research should incorporate more sensitive cognitive assessments and consider mediation analyses to explore the role of social isolation in hearing–cognition pathways.

Interpretation of these findings should also consider sample characteristics. While broadly representative of community-dwelling older adults in England and Wales, the sample may not reflect individuals with concurrent hearing loss, cognitive decline, and social isolation. If levels of cognitive impairment and isolation are low, there may be insufficient variability to detect associations. Future studies should adopt inclusive recruitment strategies and longer follow-up intervals to capture a wider range of outcomes and reduce attrition bias (31).

Biological and psychosocial mechanisms remain important considerations. Age-related hearing loss has been shown to affect cognition in a domain-specific manner, with effects varying across dementia subtypes. Animal studies indicate that prolonged moderate hearing loss can impair working and recognition memory, although the severity and pattern of deficits likely depend on the memory domain and compensatory mechanisms (32). Hearing loss and social isolation also share a complex, bidirectional relationship, whereby reduced auditory input can lead to social withdrawal, and isolation may exacerbate cognitive decline. This interplay is further complicated by central auditory processing disorders, which have been linked to neuropsychiatric outcomes, such as late-onset depression (33). These findings highlight the multifactorial nature of hearing-related cognitive decline and the need for integrated approaches addressing both auditory and psychosocial factors.

Interestingly, worse hearing was weakly associated with lower social isolation, contrary to theoretical expectations. Given the wide confidence intervals, this may reflect random variation. Alternatively, cohort-specific factors, such as strong family or community support, may buffer against isolation. These findings underscore the complexity of social dynamics and warrant further investigation. The 10-year interval between assessments may also have obscured more proximal or dynamic associations between hearing and social/cognitive outcomes. Future studies should consider repeated measures and shorter follow-up intervals to capture time-varying processes and potential non-linear effects. Alternative modeling approaches, such as examining changes in hearing over time or exploring threshold effects, may yield different insights.

Although our findings were null, they contribute to ongoing debates regarding the mechanisms linking hearing and cognition (34). The absence of associations in this relatively healthy cohort may suggest a threshold effect, whereby only more severe hearing loss impacts cognition, or that resilience factors buffer against decline. These results align more closely with the shared neuropathology hypothesis than the cascade model, though measurement limitations temper strong conclusions.

This study has several strengths, including the use of objective hearing measures via pure-tone audiometry (35) and a validated instrument (MOS Social Support Survey) to assess social isolation, avoiding reliance on proxy indicators such as marital status or living arrangements. However, limitations include potential selection bias due to attrition, modest hearing changes over time, and the use of outcome measures that may lack sensitivity for detecting subtle changes (36). More frequent follow-ups and comprehensive cognitive batteries would enhance future research. Also, we did not calculate additional effect size indices such as ΔR^2^, which could further clarify the contribution of hearing thresholds beyond covariates; future study should report these alongside regression coefficients to strengthen the interpretation.

In conclusion, although this study did not identify statistically significant associations between hearing loss and cognitive decline or social isolation, the confidence intervals suggest that small but potentially meaningful effects cannot be excluded, and the direction of estimates was consistent with prior research. Our findings emphasize the importance of sensitive outcome measures, robust sample sizes, and inclusive study designs. As populations age, integrated strategies that promote hearing health and social engagement may help support cognitive resilience in older adults.

## Data Availability

The data analyzed in this study is subject to the following licenses/restrictions: Available from MRC Lifecourse and Epidemiology, University of Southampton. Requests to access these datasets should be directed to MRC Lifecourse and Epidemiology, University of Southampton.

## References

[1] Beck D Bant S Clarke N. Hearing loss and cognition: a discussion for audiologists and hearing healthcare professionals. J Otolaryngol ENT Res. (2020) 12:72–8. doi: 10.15406/joentr.2020.12.00459

[2] Lin FR Ferrucci L Metter EJ An Y Zonderman AB Resnick SM. Hearing loss and cognition in the Baltimore longitudinal Study of aging. Neuropsychology. (2011) 25:763–70. doi: 10.1037/a0024238, PMID: 21728425 PMC3193888

[3] Lin FR Yaffe K Xia J Xue Q-L Harris TB Purchase-Helzner E. Hearing loss and cognitive decline in older adults. JAMA Intern Med. (2013) 173:293–9. doi: 10.1001/jamainternmed.2013.1868, PMID: 23337978 PMC3869227

[4] Lindenberger U Ghisletta P. Cognitive and sensory declines in old age: gauging the evidence for a common cause. Psychol Aging. (2009) 24:1–16. doi: 10.1037/a0014986, PMID: 19290733

[5] Livingston G Huntley J Sommerlad A Ames D Ballard C Banerjee S. Dementia prevention, intervention, and care: 2024 report of the lancet commission. Lancet. (2024) 396:413–46. doi: 10.1016/S0140-6736(20)30367-6 PMC7392084 32738937

[6] Baltes PB Lindenberger U. Emergence of a powerful connection between sensory and cognitive functions across the adult life span: a new window to the study of cognitive aging? Psychol Aging. (1997) 12:12–21. doi: 10.1037/0882-7974.12.1.12, PMID: 9100264

[7] Uchida Y Sugiura S Nishita Y Saji N Sone M Ueda H. Age-related hearing loss and cognitive decline—the potential mechanisms linking the two. Auris Nasus Larynx. (2019) 46:1–9. doi: 10.1016/j.anl.2018.08.010, PMID: 30177417

[8] Gates GA Mills JH. Presbycusis. Lancet. (2005) 366:1111–20. doi: 10.1016/S0140-6736(05)67423-5, PMID: 16182900

[9] Plassman BL Langa KM Fisher GG Heeringa SG Weir DR Ofstedal MB. Prevalence of dementia in the United States: the aging, demographics, and memory study. Neuroepidemiology. (2007) 29:125–32. doi: 10.1159/000109998, PMID: 17975326 PMC2705925

[10] Ren Y Savadlou A Park S Siska P Epp JR Sargin D. The impact of loneliness and social isolation on the development of cognitive decline and Alzheimer’s disease. Front Neuroendocrinol. (2023) 69:101061. doi: 10.1016/j.yfrne.2023.101061 36758770

[11] Dhanda N Hall A Martin J. Does social isolation mediate the association between hearing loss and cognition in adults? A systematic review and meta-analysis of longitudinal studies. Front Public Health. (2024) 12:1347794. doi: 10.3389/fpubh.2024.1347794 38292910 PMC10824982

[12] Alattar AA Bergstrom J Laughlin GA Kritz-Silverstein D Richard EL Reas ET. Hearing impairment and cognitive decline in older, community-dwelling adults. J Gerontol A Biol Sci Med Sci. (2019) 75:567–73. doi: 10.1093/gerona/glz035 PMC7328194 30753308

[13] Chen L Zhou R. Does self-reported hearing difficulty decrease older adults’ cognitive and physical functioning? The mediating role of social isolation. Maturitas. (2020) 141:53–8. doi: 10.1016/j.maturitas.2020.06.011, PMID: 33036703

[14] Dhanda N Pryce H. An ethnography study exploring factors that influence social isolation in care home residents living with dementia and hearing loss. BMC Geriatr. (2023) 23:593. doi: 10.1186/s12877-023-04296-0, PMID: 37749500 PMC10518931

[15] Zavaleta D Samuel K Mills CT. Measures of social isolation. Soc Indic Res. (2017) 131:367–91. doi: 10.1007/s11205-016-1252-2

[16] Sherbourne CD Stewart AL. The MOS social support survey. Soc Sci Med. (1991) 32:705–14. doi: 10.1016/0277-9536(91)90150-B, PMID: 2035047

[17] Maharani A Pendleton N Leroi I. Hearing impairment, loneliness, social isolation, and cognitive function: longitudinal analysis using english longitudinal study on ageing. Am J Geriatr Psychiatry. (2019) 27:1348–56. doi: 10.1016/j.jagp.2019.07.010, PMID: 31402088

[18] Syddall HE Simmonds SJ Martin HJ Watson C Dennison EM Cooper C. Cohort profile: the Hertfordshire Ageing Study (HAS). Int J Epidemiol. (2010) 39:36–43. doi: 10.1093/ije/dyn275, PMID: 19131391 PMC2818638

[19] British Society of Audiology. Position statement and practice guidance auditory processing disorder (APD): British Society of Audiology; (2018). Available online at: http://www.thebsa.org.uk/wp-content/uploads/2018/02/Position-Statement-and-Practice-Guidance-APD-2018.pdf (Accessed June 2022).

[20] Folstein MF Folstein SE McHugh PR. Mini-mental state: a practical method for grading the cognitive state of patients for the clinician. J Psychiatr Res. (1975) 12:189–98. doi: 10.1016/0022-3956(75)90026-6, PMID: 1202204

[21] Urdan TC. Statistics in plain English. New York: Routledge (2011).

[22] Gallacher J Ilubaera V Ben-Shlomo Y Bayer A Fish M Babisch W. Auditory threshold, phonologic demand, and incident dementia. Neurology. (2012) 79:1583–90. doi: 10.1212/WNL.0b013e31826e263d, PMID: 23019269

[23] Mick P Kawachi I Lin FR. The association between hearing loss and social isolation in older adults. Otolaryngol Head Neck Surg. (2014) 150:378–84. doi: 10.1177/0194599813518021 24384545

[24] Hong T Mitchell P Burlutsky G Liew G Wang JJ. Visual impairment, hearing loss and cognitive function in an older population: longitudinal findings from the Blue Mountains eye Study. PLoS One. (2016) 11:e0147646. doi: 10.1371/journal.pone.0147646, PMID: 26808979 PMC4726694

[25] Fischer ME Cruickshanks KJ Schubert CR Pinto AA Carlsson CM Klein BEK. Age-related sensory impairments and risk of cognitive impairment. J Am Geriatr Soc. (2016) 64:1981–7. doi: 10.1111/jgs.14308, PMID: 27611845 PMC5073029

[26] Croll PH Vinke EJ Armstrong NM Licher S Vernooij MW Baatenburg de Jong RJ. Hearing loss and cognitive decline in the general population: a prospective cohort study. J Neurol. (2021) 268:860–71. doi: 10.1007/s00415-020-10208-8, PMID: 32910252 PMC7914236

[27] Wiley TL Chappell R Carmichael L Nondahl DM Cruickshanks KJ. Changes in hearing thresholds over 10 years in older adults. J Am Acad Audiol. (2008) 19:281–92. doi: 10.3766/jaaa.19.4.2, PMID: 18795468 PMC2802451

[28] Chien W Lin FR. Prevalence of hearing aid use among older adults in the United States. Arch Intern Med. (2012) 172:292–3. doi: 10.1001/archinternmed.2011.1408, PMID: 22332170 PMC3564585

[29] Joober R Schmitz N Annable L Boksa P. Publication bias: what are the challenges and can they be overcome? J Psychiatry Neurosci. (2012) 37:149–52. doi: 10.1503/jpn.120065, PMID: 22515987 PMC3341407

[30] Yang Y Hillebrand H Lagisz M Cleasby I Nakagawa S. Low statistical power and overestimated anthropogenic impacts, exacerbated by publication bias, dominate field studies in global change biology. Glob Chang Biol. (2022) 28:969–89. doi: 10.1111/gcb.15972, PMID: 34736291 PMC9299651

[31] Hill AB. The environment and disease: association or causation? Proceedings of the Royal Society of Medicine. Sage Publications. (1965) 58:295–300. doi: 10.1177/003591576505800503 14283879 PMC1898525

[32] Uchida Y Nishita Y Tange C Sugiura S Otsuka R Ueda H. The longitudinal impact of hearing impairment on cognition differs according to cognitive domain. Front Aging Neurosci. (2016) 8:201. doi: 10.3389/fnagi.2016.00201 27597827 PMC4992677

[33] Lozupone M Sardone R Donghia R D'Urso F Piccininni C Battista P. Late-onset depression is associated with age-related central auditory processing disorder in an older population in southern Italy. Geroscience. (2021) 43:1003–14. doi: 10.1007/s11357-020-00290-1 33128133 PMC8110676

[34] Ray J Popli G Fell G. Association of cognition and age-related hearing impairment in the english longitudinal study of ageing. JAMA Otolaryngol Head Neck Surg. (2018) 144:876–82. doi: 10.1001/jamaoto.2018.1656 30193368 PMC6233824

[35] Carl A Hohman M Cornejo J. Audiology pure tone evaluation. Treasure Island (FL): StatPearls (2022). 35593838

[36] Arevalo-Rodriguez I Smailagic N Roqué IFM Ciapponi A Sanchez-Perez E Giannakou A. Mini-mental state examination (MMSE) for the detection of Alzheimer's disease and other dementias in people with mild cognitive impairment (MCI). Cochrane Database Syst Rev. (2015) 2015:Cd010783. doi: 10.1002/14651858.CD010783.pub2 25740785 PMC6464748

